# Invited Mini-Review Research Topic: Utilization of Protoplasts to Facilitate Gene Editing in Plants: Schemes for *In Vitro* Shoot Regeneration From Tissues and Protoplasts of Potato and Rapeseed: Implications of Bioengineering Such as Gene Editing of Broad-Leaved Plants

**DOI:** 10.3389/fgeed.2022.780004

**Published:** 2022-06-29

**Authors:** Erik Andreasson, Nam Phuong Kieu, Muhammad Awais Zahid, Frida Meijer Carlsen, Lenman Marit, Sjur Sandgrind, Bent Larsen Petersen, Li-Hua Zhu

**Affiliations:** ^1^ Department of Plant Protection Biology, Swedish University of Agricultural Sciences, Lomma, Sweden; ^2^ Department of Plant and Environmental Sciences, Faculty of Science, University of Copenhagen, Frederiksberg, Denmark; ^3^ Department of Plant Breeding, Swedish University of Agricultural Sciences, Lomma, Sweden

**Keywords:** protoplast, regeneration, refreshment, *Brassica napus*, rapeseed, potato, *Solanum tuberosum*, gene editing

## Abstract

Schemes for efficient regenerationand recovery of shoots from *in vitro* tissues or single cells, such as protoplasts, are only available for limited numbers of plant species and genotypes and are crucial for establishing gene editing tools on a broader scale in agriculture and plant biology. Growth conditions, including hormone and nutrient composition as well as light regimes in key steps of known regeneration protocols, display significant variations, even between the genotypes within the same species, e.g., potato (*Solanum tuberosum*). As fresh plant material is a prerequisite for successful shoot regeneration, the plant material often needs to be refreshed for optimizing the growth and physiological state prior to genetic transformation. Utilization of protoplasts has become a more important approach for obtaining transgene-free edited plants by genome editing, CRISPR/Cas9. In this approach, callus formation from protoplasts is induced by one set of hormones, followed by organogenesis, i.e., shoot formation, which is induced by a second set of hormones. The requirements on culture conditions at these key steps vary considerably between the species and genotypes, which often require quantitative adjustments of medium compositions. In this mini-review, we outline the protocols and notes for clonal regeneration and cultivation from single cells, particularly protoplasts in potato and rapeseed. We focus mainly on different hormone treatment schemes and highlight the importance of medium compositions, e.g., sugar, nutrient, and light regimes as well as culture durations at the key regeneration steps. We believe that this review would provide important information and hints for establishing efficient regeneration strategies from other closely related and broad-leaved plant species in general.

## Introduction

CRISPR/Cas has become the most important genome editing tool for both basic research and crop improvement. The ability to regenerate shoots from edited single cells constitutes a major bottleneck when implementing precise genetic editing for crop improvement on a broader scale. The capabilities for plant regeneration vary significantly between different species and genotypes (Hill and Schaller, 2013; Melnyk and Meyerowitz, 2015; Ikeuchi et al., 2016; Zhang et al., 2018), posing constraints on micropropagation, i.e., vegetative (asexual) propagation of explants derived from shoots, root tips, leaves, cotyledons, anthers, nodes, meristems and/or embryo, and genetic transformation of crop plants (Birch, 1997). Plant multiplication through clonal vegetative propagation is in many crops imperative as it preserves existing elite or superior traits (Nadakuduti et al., 2018). For example, the complex genome of tetraploid potato contains an extreme prevalence of single-nucleotide and even-length polymorphisms between alleles (Potato Genome Sequencing Consortium et al., 2011; D’Hoop et al., 2014) underlying the genetic basis for the important traits of elite cultivars (Johansen et al., 2019, Carlsen et al., 2022).

CRISPR/Cas delivery via CRISPR vectors or DNA-free ribonucleoprotein (RNP) complexes into plant cells is based on tissue culture for most plant species. The ability to induce shoot formation *in vitro* is highly variable among the species, genotypes, and accessions which are largely genetically based and constitutes a key limiting step for successful gene editing (Zhu and Welander, 2000; Zhu et al., 2001; Li et al., 2009; Burbulis et al., 2010; Ivarson et al., 2013; Farooq et al., 2019; Bidabadi and Jain, 2020; Li et al., 2021). Broad-leaved plants are generally easier to regenerate and, thus, relatively more amendable for bioengineering than for example cereals.

Apart from the genetic variation of mother plants, the age and physiological status of initial plant materials are also crucial for successful *in vitro* shoot regeneration. The leaf explants of poor *in vitro* vigor or older *in vitro* starting material, for example, are very susceptible to damage during handling as evidenced by a high and rapid necrosis incidence during the regeneration process (Birch, 1997). In general, the regeneration capacity of explants from juvenile plants is much greater than mature plants due to the ability of responsiveness to plant growth regulators (PGR) (Bidabadi and Jain, 2020). Therefore, the young leaves are mostly used for the protoplast extraction of potatoes (Moon et al., 2021). Moreover, the plant lines that have been maintained for an extended period *in vitro* tend to confer a reduced vitality as they acclimatize to *in vitro* conditions. The high humidity present within the culture vessels, a constant source of exogenous sugars, and the extended exposure to PGRs might induce some epigenetic changes that are reflected by a general decline in growth vigor (Smýkal et al., 2007; Us-Camas et al., 2014). This problem might be extra important in potatoes since clone banks are usually maintained by *in vitro* propagation for this crop.

The shoot regeneration frequencies are also highly dependent on culture conditions. For protoplast cultures, the shoot regeneration process can be divided into three major stages, namely, 1) cell wall formation, 2) callus formation and shoot induction, and 3) shoot formation. Different basal media, PGRs, and culture durations are required in each stage, of which PGRs play a crucial role. Callus formation and shoot generation are for many plant species two crucial and limiting steps in shoot generation. Many factors influence callus formation and shoot induction and formation, including genotype, explant age and type, medium composition (nutrients, type and concentration of sugars, gelling agent, type and combination of PGRs, and additional additives, e.g., silver nitrate (Pawlicki and Welander, 1994; Akasaka-Kennedy et al., 2005; Ben Ghnaya et al., 2008; Li et al., 2011; Roh et al., 2012; Li et al., 2013; Li et al., 2021). The cultural conditions such as temperature, duration, illumination quantity, quality could also have a significant effect on shoot regeneration (Akasaka-Kennedy et al., 2005; Afshari et al., 2011; Bidabadi and Jain, 2020). Moreover, the choice of CRISPR/Cas delivery system, whether being PEG, *Agrobacterium*, or ballistic bombardment, influences subsequent shoot regeneration differently (Wang et al., 2005; Zhang et al., 2005).

This mini-review outlines shoot regeneration schemes and protocols for the broad-leaved crops potato and rapeseed from single protoplast cells or tissues. The key factors affecting the callus formation and shoot regeneration are highlighted. The optimized regeneration schemes and notes for the species may provide a beneficial framework for developing efficient regeneration protocols in other species or genotypes for genome editing.

## Refreshment of Potato Explant Material

**FIGURE 1:**
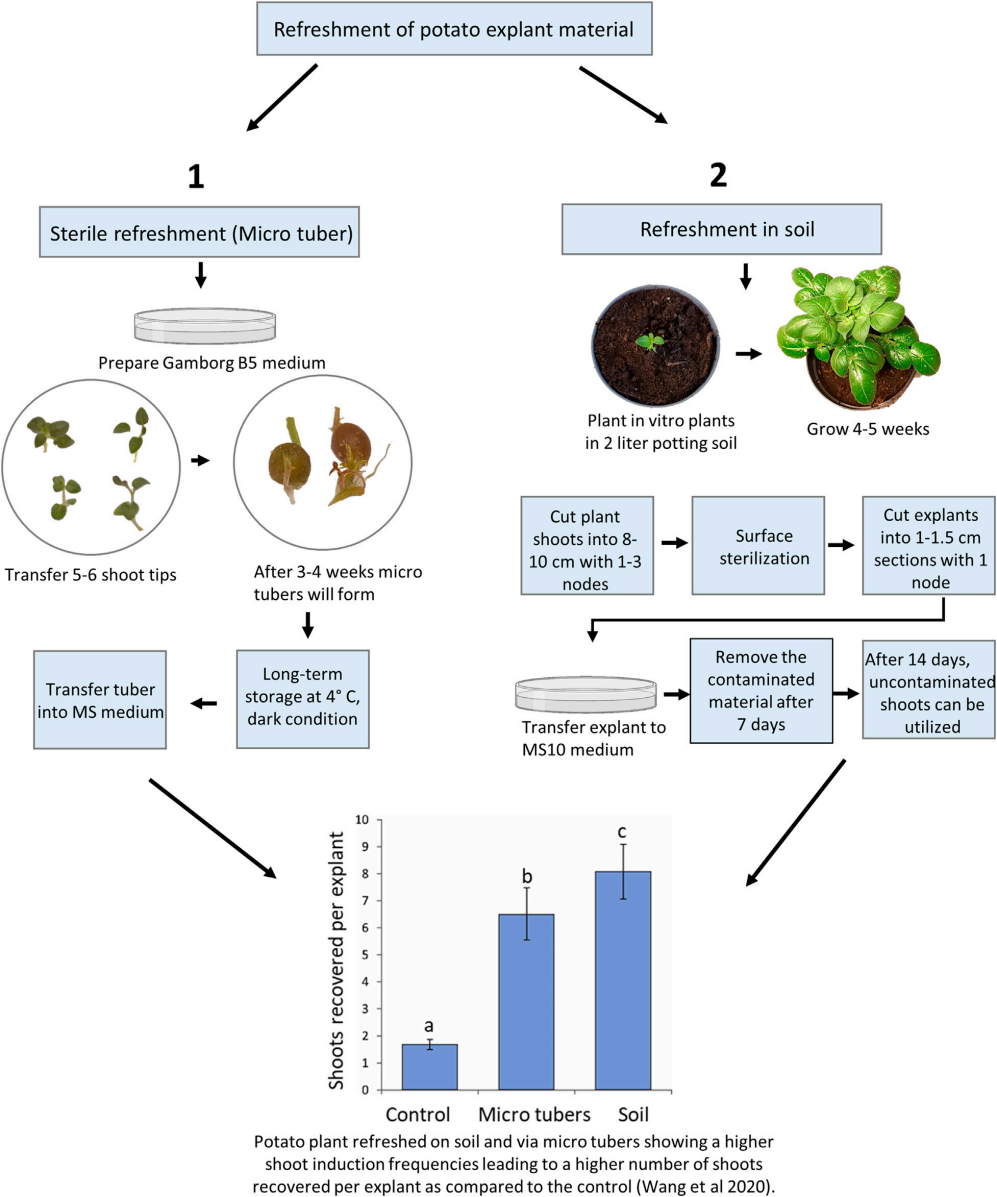
Overview of potato material refreshment. The treatments denoted by different lowercase letters indicated a significant difference at *p* < 0.05 by Tukey’s Honest Significant Difference test. The apical portion of control lines was cultured every 3–4 weeks on standard Murashige and Skoog (MS) media plus 0.01 mg/l IBA and kept at 20°C, 16 h photoperiod with 40–60 μmol/m^2^/s. A total of sixty explants from approximately 10 *in vitro* plants were observed for each treatment, and the experiments were repeated twice.

As noted above, old material maintained *in vitro* for longer periods can be difficult to regenerate and the use of refreshed plant material, e.g., potato leaf material, has been shown to improve shoot regeneration efficiencies (thus significantly improving the shoot regeneration rates in the following gene editing work (Wang et al., 2020; Kieu et al., 2021b). For potatoes, there are sterile and unsterile (soil) means of refreshment of old material with low regeneration capacities (Figure 1). Sterile refreshment is conducted by induction of mini tubers and unsterile refreshment is carried out by transferring *in vitro* plants into the soil, where they are grown for some weeks and then sterilized (Wang et al., 2020).

### Long-Term *In Vitro* Storage of Potato Material

The potato material may be stored *in vitro* for longer periods when maintained under special long-term conditions, such as the one outlined here (Kieu et al., 2021a) which is suggested to reduce the loss of regeneration capacity during the storage:

1. Prepare 60 × 15 mm Petri plates with 10 ml MS medium (4.4 g/l MS salt including vitamins, 10 g/l sucrose, 8 g/l agar, pH 5.8 ± 0.1). This will confer a more solid medium and dry surface, which is likely to reduce contamination and hyperhydricity (also known as “vitrification”).
2. Transfer two to three shoots into a Petri dish. Seal the plate with Parafilm and incubate for 4 weeks in a normal growth chamber with the growth conditions: 20°C, 16 h photoperiod with 40–60 μmol/m^2^/s.
3. Place the plates in a 9°C, 16-h photoperiod, with 20–40 μmol/m^2^/s. The *in vitro* potato lines may now be stored for up to 6 months. In order to reduce the risk of losses due to power failure, two Petri dishes for each line are made and kept at different places.

Cryopreservation is an alternative way of establishing long-term *in vitro* storage, which may be challenging to implement and standardize for small laboratories from our experience.

### Sterile Refreshment of Potato Material

The potato *in vitro* stock lines is stored at low temperatures as described above to reduce plant growth. After long-term storage, some stock lines may not be optimal material for *Agrobacterium* transformation or protoplast transfection as they downstream tend to result in reduced shoot regeneration frequencies. Recently, the refreshment protocols were quantitatively compared with the most optimal displaying significantly improved growth vigor and resulting in a 4 to 10-fold increase in transformation efficiency (Wang et al., 2020). The sterile method was almost comparable to the soil-based method (Figure 1), and in agreement with the findings in *S. tuberosum* cv. Atlantic (Han et al., 2015) where “fresh explants” were prepared using a similar *in vitro* method. Furthermore, long-term exposure to exogenous Indole-3-butyric acid which is usually used for the initiation of roots *in vitro* was found to cause aberrant morphological phenotypes in potatoes (Wang et al., 2020). Thus, a periodic refreshment of *in vitro* lines is highly beneficial for increasing plant consistency, vigor, and ultimately editing success in regenerated explants.

*In vitro* mini tuber is a good material for long-term storage of stock lines and refreshment of stock lines (Han et al., 2015; Wang et al., 2020). In nature, besides having a nutrient storage function, the potato tuber provides means for asexual reproduction. The potato tuber contains nutrient and bud dormancy to help the potato plant pass winter conditions and induce fast growth in the spring. *In vitro* mini tubers can be produced using the following procedure and used for producing refreshed plant material for protoplast isolation and transformation.

1. Prepare 90 × 25 mm Petri plates with 30 ml Gamborg B5 medium (3.2 g/l Gamborg B5 salt including Vitamins, 80 g/l sucrose, 5 g/l Gelrite, or 8 g/l agar, pH 5.8 ± 0.1).
2. Transfer five to six shoot tips into each plate. Seal the plate with micropore medical sealing tape. Put the culture in the dark in a growth chamber at 20 C.
3. After 3–4 weeks micro tubers will be formed and then remove the micropore medical sealing tape and seal the plate with Parafilm instead. Keep at 4°C, in darkness.
4. After two to 3 years, the potato lines should be renewed by culturing in MS medium containing 1% sucrose for 4 weeks in a growth chamber before induction of micro tuber for a second round of *in vitro* long-term storage.
5. After long-term storage most mini tubers have white and long shoots. Shoots or mini tubers with dormant buds should be transferred into a new medium and kept in a growth chamber (20°C, 16 h photoperiod, and 40–60 μmol/m^2^/s), and after 2 weeks new refreshed small shoots will be formed.

Nitrogen, carbohydrate, light, and temperature are the factors that induce tuber formation in potato (Jackson, 1999; Fischer et al., 2008). The Gamborg B5 medium was used to induce *in vitro* mini tubers because the nitrogen concentration is lower than in the MS medium. Low nitrogen levels in combination with a high concentration of the carbon source (sucrose) stimulate mini tuber production. Darkness is also recommended for the induction of mini tubers (Kieu et al., 2021a).

### Refreshment in Soil of Potato Material

We have previously shown that the *ex vitro* (in soil) method for the refreshment of plant material provides more efficient shoot regeneration than the *in vitro* mini tuber refreshment method (Wang et al., 2020). This method follows the transfer of *in vitro* plants into the soil, where they are growing for 4–5 weeks and re-entered into the *in vitro* scheme (Kieu et al., 2021a):

1. Put *in vitro* plants in 2 L pots (Φ 16.7) with soil and grow for 4–5 weeks in a growth chamber (20°C, 16 h photoperiod, and 160 μmol/m^2^/s with fluorescent lamps, relative humidity 65%).
2. Cut plant shoots into 8–10 cm explants each carrying 1 to 3 nodes. Remove all leaves and put up to 20 explants in a 500 ml Erlenmeyer flask. Wash the explants 3 times with water, 1 time with 1% dishwasher solution, and 3 times after this with water to remove all dishwasher solution.
3. In a laminar airflow cabinet, wash the plant material one time with sterile water, followed by shaking for 45 s in 200 ml 70% ethanol. Wash directly with 200 ml 4% NaOCl containing 3 to 4 drops of Tween^®^20 for 5 min. Finally, wash several times with sterile water until all NaOCl has been removed.
4. Remove dead de-colored or white tissue and cut the explants into 10–15 mm sections each containing one node. Transfer each explant into a 60 × 15 mm Petri dish containing 10 ml MS10 medium (containing 1% sucrose and 8% agar). Seal the plates with a micropore medical sealing tape and keep them in the tissue culture room (20°C, 16 h photoperiod, 40–60 μmol/m^2^/s).
5. After 7 days, remove the contaminated material.
6. After 14 days, non-contaminated shoots can be used as *in vitro* material.

Although this method increase shoot recovery compared to the sterile method (Figure 1), it has some disadvantages, including a longer overall time span, requirement of plant growth chambers, and surface sterilization of the plant material, which is not always successful because it is highly dependable on the microorganism community on plant surface (Misra and Misra 2012). In addition, the explants of plants grown in the open field are more prone to contamination than plants grown in growth chambers and therefore, they need a higher concentration of the sterilizing agent NaOCl or increased treatment time.

## Protoplast Regeneration and Transfection of Rapeseed

**FIGURE 2:**
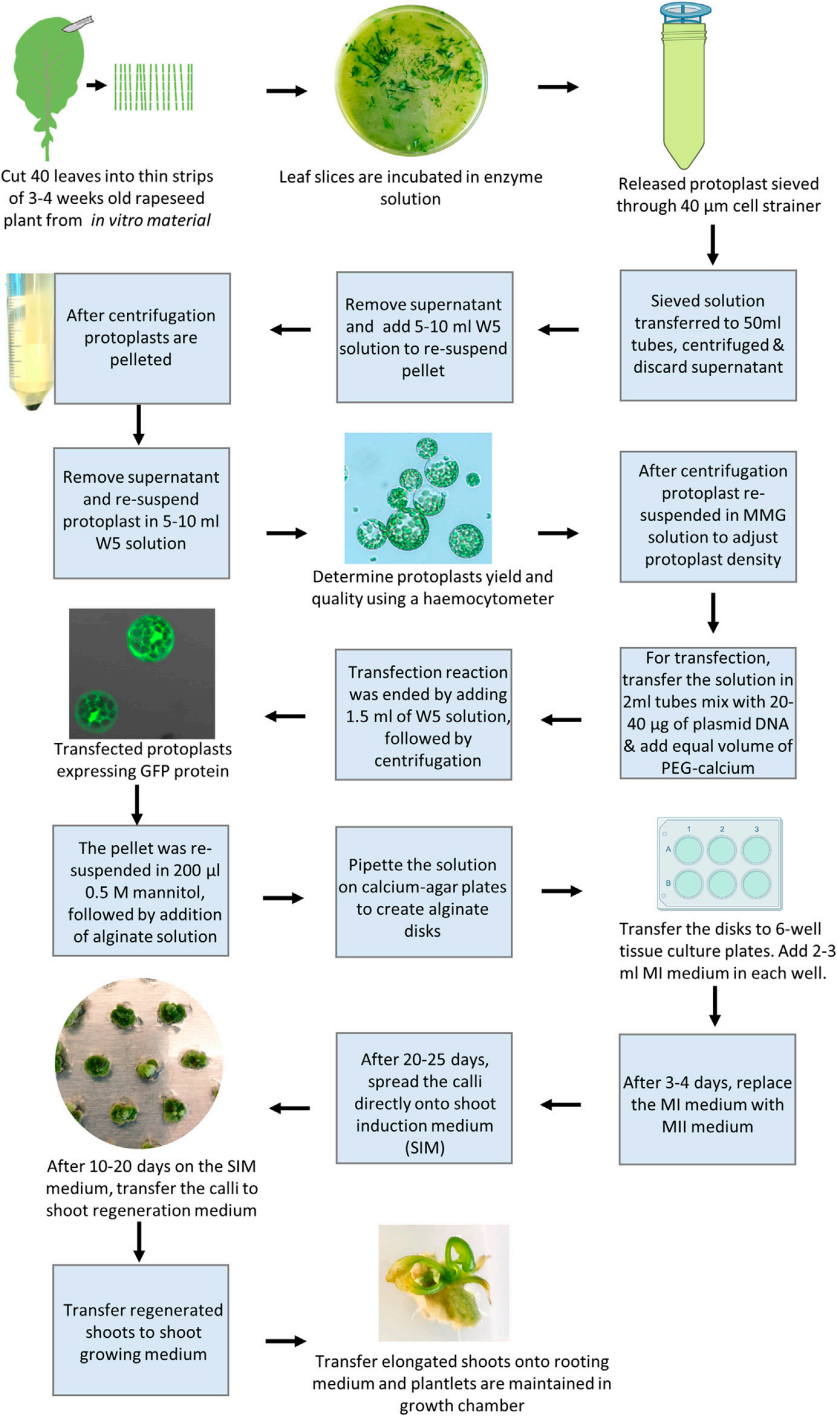
Overview of protoplast regeneration and transfection of rapeseed (*Brassica napus*)

CRISPR/Cas9 edited mutation lines of rapeseed have been obtained recently through stable *Agrobacterium*-mediated transformation of hypocotyls (Braatz et al., 2017; Li et al., 2018; Huang et al., 2020; Zheng et al., 2020), normally resulting in T-DNA insertion in the genome. The mutated line may be backcrossed to get rid of the T-DNA insertion and thus obtain a transgene-free mutation line where after homozygosis with respect to the mutations, can be restored, an iterative process requiring a number of generations due to tetraploidy of rapeseed. PEG-mediated protoplast gene editing would thus be desirable as it would enable direct generation of transgene-free explants. The complicated protoplast regeneration in rapeseed has been the major limiting factor for establishing transgene-free gene editing (Li et al., 2021), and attempts to generate gene-edited mutated lines using PEG-mediated protoplast transformation which have until recently been unsuccessful (Lin et al., 2018; Murovec et al., 2018). However, recently a breakthrough was published by Li et al. (2021). As stated in the introduction a number of factors affect protoplast regeneration, including the type of basal media, type, and concentration of sugars, concentration and combination of PGRs, age and type of explants, and culture duration at different stages of protoplast development. By taking these factors into consideration, we have recently developed an efficient protoplast regeneration and transfection protocol for rapeseed cv. Kumily. Using this protocol, we were able to successfully generate CRISPR edited lines of rapeseed, with reasonable mutation and regeneration frequencies (Li et al., 2021). Following, we summarized some important points in this protocol and presented the protocol stepwise as well as visualize it (Figure 2).

Different media are required in each stage, in which PGRs play a crucial role in the shoot regeneration. The cell wall formation is the first crucial step of protoplast culture, which starts within a few hours after isolation and may take several days to complete (Kartha et al., 1974). During this stage, a high concentration of auxin is required to induce the cell wall formation, particularly in the presence of a high level of 2, 4-D, while no addition of cytokinin in the medium is needed (Glimelius, 1984; Li et al., 2021). Once the cell wall has formed, protoplasts enter the next crucial developmental stage, i.e. callus formation and shoot induction. During this stage, the auxin levels need to be reduced while a relatively high level of cytokinin is required for promoting first callus formation and then shoot induction. Our results have shown that thidiazuron (TDZ) was the most effective cytokinin source for shoot induction. The next important stage is shoot formation, which requires an optimal balance of cytokinin and auxin and the combination of TDZ and NAA is shown to be the best among all the combinations tested (Li et al., 2021).

To provide osmotic protection for the protoplasts (Kao and Seguin-Swartz, 1987), mannitol is crucial in the media during the first 40 days of protoplast culture, while a higher concentration is needed only in the first 3–5 days, thereafter the mannitol level needs to be reduced to half in order to maintain normal callus growth and shoot induction, and to be removed completely during shoot formation (Li et al., 2021). Moreover, strict control of culture duration(s) at different stages is a prerequisite for achieving a satisfactory shoot regeneration frequency (Li et al., 2021).

The age of starting material or seedlings is important for obtaining a high yield and robust protoplasts. We found that leaves from 3 or 4-week-old rapeseed seedlings conferred a high protoplasts yield. The leaves older than 4 weeks could be used, but the protoplast yield was lower. It has been reported that regeneration can be induced from protoplasts isolated from hypocotyls or leaves of seedlings (Poulsen and Nielsen, 1989; Li et al., 2021). According to our experience, it is more practical and productive to work with leaves and achieve sufficient regeneration frequency after transfection (Li et al., 2021). It is also important to point out that the size of protoplast calli, according to our experience, should be as small as possible, ca. 1–2 mm in diameter, before being transferred onto the shoot induction medium (Li et al., 2021), while others have stated that protoplast colonies should not be less than 2 mm in diameter before transfer (Barsby et al., 1986; Poulsen and Nielsen, 1989).

Below is the procedure of the optimized protocol for rapeseed protoplasts along with some notes (see also Figure 2):

1. Use fully opened true leaves from 3 to 4 weeks old seedlings, grown *in vitro* on half-strength MS medium, 10 g/l sucrose, 7 g/l Bacto agar at pH 5.7 as start material.
2. Cut about 40 leaves quickly into thin strips on wetted filter paper with a razor blade and incubate them in plasmolysis solution (0.4 M mannitol at pH 5.7) for 30 min at room temperature (RT) in a Petri dish.
3. Replace the plasmolysis solution with 10 ml freshly prepared enzyme solution (1.5% (w/v) cellulase Onozuka R-10 (Yakult Pharmaceutical Co., LTD., Tokyo, Japan), 0.6% (w/v) Macerozyme R-10 (Yakult Pharmaceutical Co., LTD.), 0.4 M mannitol, 10 mM MES, 0.1% (w/v) BSA, 1 mM CaCl_2_, and 1 mM *β*-mercaptoethanol at pH 5.7), and incubate it in darkness with gentle shaking (ca. 25 rpm) for 14–16 h.
4. Add 10 ml W5 solution (Menczel et al., 1981) and incubate further in the dark with gentle shaking for 10 min.
5. Filter the solution through a 40 μm cell strainer. Add 20 ml W5 into the Petri dish to rinse the Petri dish, and filter the solution through the same cell strainer.
6. Centrifuge the solution at 100 x *g* for 10 min, with brake and acceleration set to a minimum.
7. Remove the supernatant and add 10 ml W5 to resuspend the pellet with gentle rocking. Centrifuge the solution at 100 *g* for 5 min and repeat this step twice.
8. Add 5 ml W5 and incubate on ice for 30 min.
9. Remove the supernatant and resuspend the pellet in 5 or 10 ml W5, depending on the size of the pellet. Use 15 µl of the solution to estimate the protoplast yield and quality on a Hemocytometer under a light microscope. A common yield is about 600,000–800,000/ml.
10. Centrifuge the solution at 100 x *g* for 3 min, and adjust the protoplast density to 400,000 to 600,000 protoplasts per ml using freshly prepared MMG solution (0.5 M mannitol, 15 mM MgCl_2_, and 4 mM MES). Note: The culture density of protoplasts is an important factor affecting the protoplast growth and development.
11. Transfer the solution in 200 µl aliquots in 2 ml eppendorf tubes, and gently mix with 20–40 µg plasmid DNA (1 μg/μl) and an equal volume of freshly prepared PEG-calcium solution (25% (w/v) PEG 4000, 0.5 M mannitol, and 0.1 M CaCl_2_).
12. Stop the reaction after 5 min by adding ∼1.5 ml W5, followed by gentle inversion of the tubes and centrifugation at 100 *g* for 3 min.
13. Remove the supernatant and resuspend the pellets in 200 µl 0.5 mannitol, followed by the addition of 200 µl alginate solution (2.8% (w/v) sodium alginate and 0.4 M mannitol).
14. For the formation of alginate disks, pipette the protoplast solution onto the calcium-agar plates (0.4 M mannitol, 2.2 g/l CaCl_2_, and 10 g/l Phyto agar) and incubate it at RT for 30 min. Thereafter, add ∼2 ml calcium solution (50 mM CaCl_2_ and 0.4 M mannitol) onto each disk, and incubate the disks for 1 h to complete polymerization.
15. Transfer the disks to 6-well tissue culture plates (one disk/well). Add 2–3 ml MI medium (2.18 g/l Nitsch medium, 10 g/l sucrose, 10 g/l glucose, 100 g/l mannitol, 100 mg/l caseium, 0.5 mg/l NAA, and 0.5 mg/l 2, 4-D at pH 5.7) in each well. Keep the plates in the dark at RT for 24 h. Afterward, cover the plates with fiber cloth and keep them in a climate chamber with a temperature of 23°C/18°C (day/night) and 16 h photoperiod with a light intensity of 40 μmol m^2^ s^1^ (cool white fluorescent tubes).
16. After 3–4 days, replace the MI medium with an MII medium (2.18 g/l Nitsch medium, 10 g/l sucrose, 10 g/l glucose, 100 g/l mannitol, 100 mg/l caseium, 1.1 mg/l TDZ, and 0.05 mg/l 2,4-D at pH 5.7). Refresh the MII medium every 5–7 days. *Note: A prolonged culture duration at this step would reduce regeneration rapidly.*
17. After 20–25 days, spread the calli directly onto the shoot induction medium (SIM) (MS, 30 g/l sucrose, 50 g/l mannitol, 2.2 mg/l TDZ, 0.05 mg/l NAA, 0.5 mg/l AgNO_3_, and 2.5 g/l Gelrite at pH 5.7). Note: a prolonged culture duration at this step would reduce regeneration rapidly.
18. After 10–20 days on the SIM medium, transfer the calli to the shoot regeneration medium (MS, 20 g/l sucrose, 0.5 mg/l AgNO_3_, 2.2 mg/l TDZ, 0.5 mg/l NAA, and 2.5 g/l Gelrite at pH 5.7). Subculture the calli with fresh medium every 3–4 weeks.
19. Transfer the regenerated shoots to the shoot growing medium (MS, 20 g/l sucrose, 0.05 mg/l BAP, 0.03 mg/l GA_3_, and 7.5 g/l Bacto agar at pH 5.7)
20. Transfer the elongated shoots onto the rooting medium (MS, 20 g/l sucrose, 0.05 mg/l NAA, and 7.5 g/l Bacto agar at pH 5.7), and plantlets are maintained in a growth chamber or greenhouse and ready for further analysis.

Finally, it should be kept in mind that the abovementioned optimized protocol may need to be adjusted for other or new genotypes of rapeseed. It has been reported that the genetic variation in regeneration capacity was greater for *Brassica napus* cultivars than the variation reported between different species within the *Brassica genus*. Comparing the pedigrees of cultivars from high- and low-regenerating cultivars did not reveal any simple correlation between the taxonomical relatedness and the growth and regeneration ability in the protoplast culture (Poulsen and Nielsen, 1989).

## Regeneration From Protoplasts of Potato

The efficiencies of potato regeneration can be optimized by improving the quality of the starting plant material (see above), and other factors of importance for the regeneration steps such as optimized media for each genotype (Table 1). We have earlier established gene editing in several tetraploid potato varieties and generated full allelic edited plants derived either from the transformation of CRISPR/Cas constructs delivered by Agrobacterium (integrative) to leaves (Kieu et al., 2021b) or Poly Ethylene Glycol (PEG) (transient non-integrative) to protoplasts (Johansen et al., 2019). We have also optimized gene editing efficiency at the protoplast level (Johansen et al., 2019; Petersen et al., 2019) and editing scoring (Kemp et al., 2017; Johansen et al., 2019; Bennett et al., 2020). A three to four-fold increase in the editing efficiency at the protoplast level, yielding editing in >50% editing of all alleles in the cell pool, was obtained by replacing the standard *A. thaliana At*U6-1 promotor with endogenous *St*U6 promotors driving expression of gRNAs targeting the granular bound starch synthase (GBSS) 1 gene, with (Johansen et al., 2019). Lately, we mapped the editing efficiency in relation to gene structure and placement of CRISPR/gRNA-targeting, i.e. start versus end, of a gene, and the effect of applying several RNPs (multiplexing) targeting the same gene versus targeting genes residing on different chromosomes (Carlsen et al., 2022). Although CRISPR derived off-target events have attained some focus, unintentional small genetic changes (somaclonal variation) associated with cell and tissue culture e.g. from a single protoplast cell, have been shown to be significantly higher than mutations from CRISPR derived off-target events (Tang et al., 2018; Li et al., 2019). The extended protoplast regeneration periods and repeated rounds of tissue culturing should thus ideally be avoided.

**TABLE 1:** Media Composition for the maintenance, transformation, selection, and regeneration of the potato genotypes Désirée, King Edward, and B101 (diploid). CIM = callus induction media; SIM = shoot induction media.

Component	Plant material			Use
	Désirée	King Edward	B101 (diploid)	
MS1				Maintenance of *in vitro* lines, rooting media, and stock propagation
MS salts + vitamins	4.4 g/l	4.4 g/l	4.4 g/l	
Sucrose	10 g/l	10 g/l	10 g/l	
pH	5.8	5.8	5.8	
Phytoagar	8 g/l	8 g/l	8 g/l	
CIM (MS1 including compounds below)				Co-cultivation post transformation, callus induction
BAP	2.0 mg/l	2.0 mg/l	2.0 mg/l	
trans-Zeatin-riboside	—	—	—	
NAA	0.2 mg/l	0.2 mg/l	0.2 mg/l	
GA_3_	—	—	—	
SIM (MS1 including compounds below)				Selection and regeneration post transformation, shoot induction
BAP	—	—	—	
trans-Zeatin-riboside	2.0 mg/l	**4.0 mg/l**	2.0 mg/l	
NAA	0.01 mg/l	0.01 mg/l	—	
GA_3_	0.1 mg/l	0.1 mg/l	**2.5 mg/l**	
Kanamycin	100 mg/l	100 mg/l	100 mg/l	
Claforan (cefotaxime)	400 mg/l	400 mg/l	400 mg/l	

In bold are major optimizations suggested by Wang et al 2020.

**FIGURE 3:**
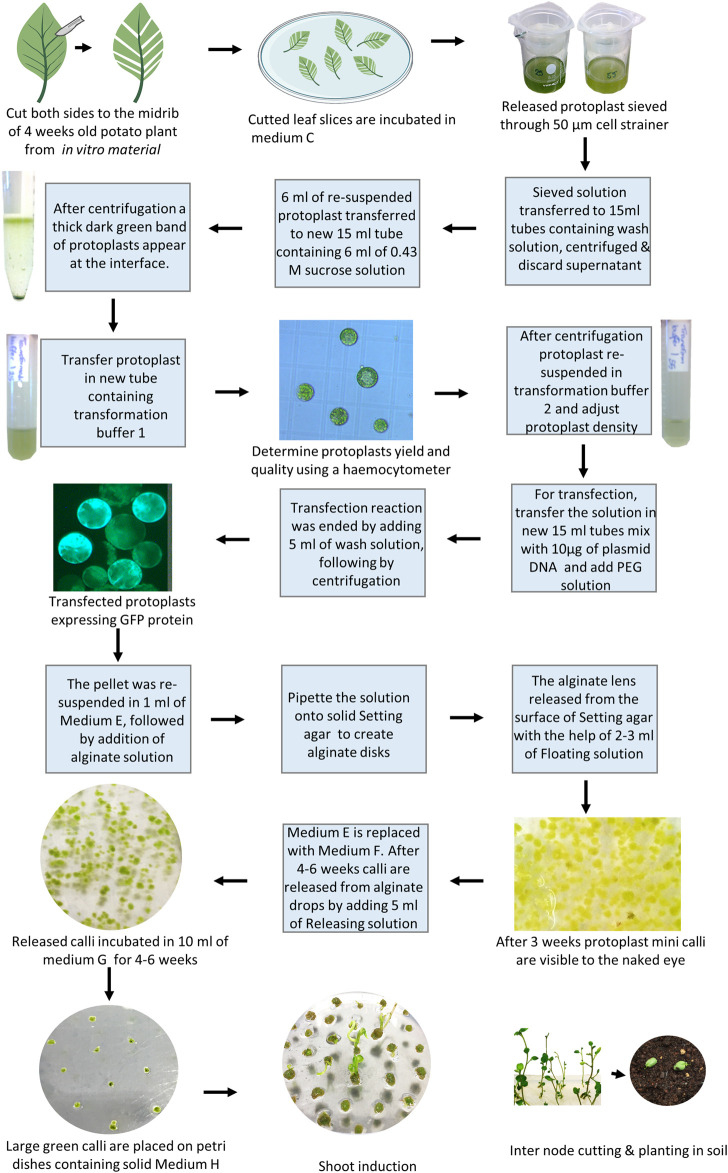
Overview of protoplast and transfection in potato.

Here, we present and comment on a successful regenerating protocol from gene-edited potato protoplasts to shoots (Figure 3), mainly based on our method published by Nicolia et al. (2015) and with recent adjustments as described in Moon et al., 2021.

1. Maximum yield of protoplasts has been achieved using leaflets from 4-week-old *in vitro* material by cutting both sides from the midrib (using leaf strips with midrib), thus reducing wounding to a minimum (Moon et al., 2021). Finely cut leaf slices are incubated in medium C containing 1% (w/v) cellulase and 0.5% (w/v) Macerozyme for 18 h in complete darkness (Moon et al., 2021). *Note: Gentle shaking at 40 rpm in the darkness can increase the protoplast yield substantially.*
2. Two sterile filters of 100 and 70 µm are mounted together on a 50 ml tube and pre-wetted with 5 ml of wash solution (Supplementary material).
3. The solution containing released protoplasts is gently aspirated with a pipette and sieved through the filters, the remaining protoplasts are washed from the filters using 30 ml of wash solution (i.e., 3x more washing buffer than the amount normally used increased the yield (Moon et al., 2021).
4. The suspension with the sieved protoplasts is transferred to sterile 15 ml centrifuge tubes (8 ml per tube), and the tubes are filled to 15 ml with an additional wash solution. After a 5 min centrifugation at 50 *g*, with minimum acceleration and deceleration, the supernatant is discarded and protoplasts are gently resuspended in 2 ml of wash solution.
5. New sterile 15 ml centrifuge tubes, each containing 6 ml of 0.43 M sucrose solution are prepared and a maximum of 6 ml of resuspended protoplasts is slowly layered on top with a sterile Pasteur pipette, without disrupting the interface. The tubes are subsequently centrifuged at 50 x *g* for 15 min (with minimum acceleration and deceleration). A thick dark green band of protoplasts should appear at the interface.
6. Add 3 ml of transformation buffer 1 into a new sterile 15 ml centrifuge tube. Using a pipette with a cut tip, the protoplasts are gently transferred into the tube. Ten microliters of protoplasts is used to determine the density (protoplasts/ml) using a hemocytometer. The protoplasts can be stored in transformation buffer 1 at 4°C in darkness until counting.
7. The protoplasts are centrifuged at 50 x *g* for 10 min (minimum acceleration and deceleration), the supernatant is subsequently discarded, and the protoplasts are gently resuspended in transformation buffer 2 at the concentration of 1.6 × 10^6^ protoplasts/ml.
8. Fresh sterile 15 ml centrifuge tubes are prepared for each transfection or control. Ca 10 µg of plasmid DNA (10–20 µl) or ribonucleoprotein (RNP) (for amount and incubation see manufacturer instructions and Carlsen et al., 2022) are pipetted in each tube followed by 100 µl of protoplasts in transformation buffer 2that equals approximately 160.000 protoplasts.
9. A volume ranging from 110 to 120 µl of PEG solution, accordingly to the volume of plasmid DNA used, is gently added to each tube (tubes are gently flicked before and after adding the PEG solution). The samples are incubated at RT for 3 min.
10. Transfection reactions are ended by carefully adding 5 ml of Wash solution to each tube and subsequently centrifuged at 50 x *g* for 5 min (with minimum acceleration and deceleration).
11. The supernatant is discarded and the pellet is gently resuspended in 1 ml of medium E. The same volume of alginate solution is added to give a final density of 8 × 10^4^ protoplasts/ml (corresponding to 2 × 10^3^ protoplast lens to allow continuous development, Moon et al., 2021). The two solutions are gently mixed inverting the tubes and the solution is transferred in aliquots (usually 4 big drops to form 4 disks) to the surface of solid setting agar in a regular 90 mm Petri dish. The disks are left at RT for a maximum of 2 h to allow solidification.
12. The alginate lens is subsequently released from the surface of Setting agar with the help of 2–3 ml of Floating solution and moved to fresh Petri dishes containing 10 ml of Medium E. Petri dishes are sealed with Parafilm, covered with aluminum foil and incubated at 25°C for 3 days.
13. After 3 days the light is gradually increased by replacing the aluminum foil with a paper towel (kitchen paper). Once protoplast mini calli are visible to the naked eye (usually after 3 weeks), medium E is replaced with 10 ml of medium F. The calli can be exposed to full light (40–60 μmol/m^2^/s) at this stage. Change medium F every week.
14. After 4–6 weeks in medium F, calli are released from alginate drops by adding 5 ml of Releasing solution and incubating for a maximum of 10 min. A forceps or a pipette tip can be gently used to help the release of the calli. The releasing solution is carefully aspirated and the calli washed with 10 ml of medium F. The released calli are then incubated in 10 ml of Medium G for the other 4–6 weeks (fresh Medium G is provided every week). Greening of callus has an effect on regeneration efficiency because functional chloroplasts have developed in these callus cells and they can synthesize necessary compounds for shoot regeneration.
15. Large green calli are then briefly dried on a sterile filter paper, moved individually on Petri dishes containing solid medium H, and incubated in the same conditions used for potato propagation. Calli are moved on fresh medium H each 10–15 days and the shoots usually emerge after 3 months of culture. *Note: Gelrite can be beneficial to use* (Moon et al., 2021).
16. The mature shoots are moved to solid medium I for rooting and plantlets are moved to medium A.

### Medium Compositions for Different Potato Cultivars

The hormone composition in media might need to be adjusted to the genotype of interest. As an example Table 1 shows optimized media for three different potato materials (Wang et al., 2020). Desirée is a standard cultivar and most protocols work on this genotype, but this may not be the case for other cultivars. In brief, higher levels of trans-zeatin riboside of at least 3.0 mg/l were important for the induction of buds from callus material of cultivar King Edward. After 4–6 weeks on the shoot induction media, the developed buds are readily differentiated into shoots. For B101, a diploid breeding line, the callus material was able to develop buds that remained dormant and required elevated levels of GA_3_ to promote shoot development. The optimal concentration of GA_3_ was determined to be 2.5 mg/l for the regeneration of shoots from B101.

## Conclusion and Comment

Tissue culture-based methods are commonly used for genetic transformation and gene editing in basic plant research as well as crop improvement, and the protoplast approach has a great potential for generating transgene-free CRISPR/Cas edited lines. A major challenge in most crop species is *in vitro* shoot regeneration, particularly when regenerated from the protoplasts. This is mainly because shoot regeneration varies highly between species and genotypes, age, type of explants, culture conditions, etc. In this mini-review, we summarized some important factors affecting the *in vitro* shoot regeneration from the protoplasts of potato and rapeseed, such as not prolonged periods of incubation times. The protoplast regeneration protocols for rapeseed and potato have some important common steps and factors affecting shoot regeneration (Figures 2, 3), while there are some differences, such as how to cut the leaves, filter size, and collection of protoplasts as well as medium compositions, especially PGRs. We also pointed out the importance of using fresh plant material for vegetative propagation and presented plant material refreshment schemes for the optimal physiological state of potatoes. These protocols provide new perspectives for both basic research and trait improvement of these two important crops and may provide a framework or foundation for developing regeneration schemes for other genotypes within the target species or related broad-leaved species.
