# Supplementary material for: Invited Mini-Review Research Topic: Utilization of Protoplasts to Facilitate Gene Editing in Plants: Schemes for In Vitro Shoot Regeneration From Tissues and Protoplasts of Potato and Rapeseed: Implications of Bioengineering Such as Gene Editing of Broad-Leaved Plants
Source: Front Genome Ed. 2022 Jun 29;4:780004. doi: 10.3389/fgeed.2022.780004 (PMC9276966; doi:10.3389/fgeed.2022.780004)
Supplement: Supplementary file 1 [file DataSheet1.docx]

Invited Mini-review Research Topic: [Utilization of Protoplasts to Facilitate Gene-Editing in Plants](http://links.email.frontiersin.org/ls/click?upn=AAaFa03elZRFPXQ6ShiKwAPsR8oZaO3IEOnZve2aIAUVWfbtoG-2FPraQhSbKxpKt7xPANYqvSCi9m-2FuRjqfokmQ-3D-3DfarO_A1OU-2BgvstZXzzB5-2Fu2HtAacCqBVz6M9CHPIHQXSRrJWrqUkXmEFlZgOvN1V9T059c-2F0hWyDLbgsljy7Sj8AAt4T1iwbBYDW3UcLgY1xBVvpQeG5qYlZbjE4wte1rMKnyyzTVHip-2FGlg8A-2F15DO7Y-2FLMjeMT50FVUGDYv5pHO6VY3l3iU-2FOGPIpZAtTvEV8pI7mXBt7yn1lzUOw3xIdnVOSkcZHhKbSu7gEERLJuDEI3nIdXwsDXkK-2FeSZuKe20vcwRfYjWQjjaJRWXpI0uCdKw5VJS5oXM3kjK-2BuapyCEuc-3D):

Schemes for in vitro shoot regeneration from tissues and protoplasts of potato and rapeseed: implications for bioengineering such as gene editing of broad leaved plants

**SUPPLEMENTARY MATERIAL**

**Media and solutions recipes for potato protoplast regeneration**

| **Medium A** | **For 1 L** |
| --- | --- |
| ½ MS salts and organics (Duchefa) | 2.2 g |
| Vit. MS stock | 0.5 ml |
| Sucrose | 15 g |
| Agar | 8 g |
| pH | 5.6 (KOH) |
| * filter sterilize in ½ of the volume, then add to autoclaved agar in the other half | |

| **Medium B*** | **For 1 L** |
| --- | --- |
| MS modif. No. 4 (Duchefa) | 2.7 g |
| Vitamins NN stock | 0.1 ml |
| Casein hydrolysate | 100 mg |
| NAA | 2 mg |
| BAP | 0.5 mg |
| pH | 5.8 (KOH) |
| * filter sterilize |  |

| **Plasmolysis solution*** | **For 1 L** |
| --- | --- |
| D-Sorbitol | 91.1 g (0.5 M) |
| * filter sterilize |  |

| **Medium C *** | **For 1 L** |  |
| --- | --- | --- |
| Macro stock | 10 ml |  |
| CaCl_2_ stock | 3 ml (6 mM) |  |
| Iron stock | 10 ml |  |
| Micro stock | 1 ml |  |
| Vitamin mix 1 stock | 5 ml |  |
| Vitamin mix 2 stock | 5 ml |  |
| Vitamin mix 3 stock | 5 ml |  |
| Sugars stock | 20 ml |  |
| Organic acids stock | 10 ml |  |
| Casein hydrolysate | 500 mg |  |
| Glucose | 36.95 g (0.205 M) |  |
| Mannitol | 37.35 g (0.205 M) |  |
| PVP 10 | 20 g |  |
| NAA | 1 mg |  |
| BAP | 0.4 mg |  |
| Cellulase RS (Yakult) | 10 g |  |
| Macerozyme (Yakult) | 2 g |  |
| pH | 5.6 (KOH) |  |
| * add all the components with the exception of CaCl_2_, then check the pH and incubate at 55°C for 10 min. Let to cool down to RT, add CaCl_2_ and filter sterilize. Prepare fresh. | | |

| **Wash solution*** | **For 1 L** |
| --- | --- |
| Macro stock | 10 ml |
| CaCl_2_ stock | 3 ml (6 mM) |
| Iron stock | 10 ml |
| Micro stock | 1 ml |
| NaCl | 14.03 g (0.24 M) |
| NAA | 2 mg |
| BAP | 0.5 mg |
| pH | 5.6 (KOH) |
| * filter sterilize | |

| **Sucrose solution*** | **For 1 L** |
| --- | --- |
| Sucrose | 147.2 g (0.43 M) |
| * filter sterilize | |

| **Transformation buffer 1*** | **For 250 ml** |
| --- | --- |
| Mannitol | 8.65 g (190 mM) |
| CaCl_2_ · 2H_2_O | 3.67 g (100 mM) |
| MES | 1.25 g (0.5% w/v)) |
| pH | 5.6 (KOH) |
| * filter sterilize | |

| **Transformation buffer 2*** | **For 50 ml** |
| --- | --- |
| Mannitol | 4.55 g (0.5 M) |
| MgCl_2_ · 6H_2_O | 152 mg (15 mM) |
| MES | 50 mg (0.1% (w/v)) |
| pH | 5.6 (KOH) |
| * filter sterilize | |

| **PEG solution*** | **For 10 ml** |
| --- | --- |
| PEG 4000 (Fluka) | 2.5 g (25% (w/v)) |
| Mannitol^a^ | 5 ml (0.4 M) |
| Ca(NO_3_)_2_^b^ | 500 ul (0.1 M) |
| *filter sterilize, prepare fresh  ^a^ use a 0.8 M filter sterilized stock solution  ^b^ use a 2 M filter sterilized stock solution | |

| **Medium E *** | **For 1 L** |
| --- | --- |
| Macro stock | 10ml |
| CaCl_2_ stock | 1.25 ml (2.5 mM) |
| Iron stock | 10 ml |
| Micro stock | 1 ml |
| Vit. mix 1 stock | 5 ml |
| Vit. mix 2 stock | 5 ml |
| Vit. mix 3 stock | 5 ml |
| Sugars stock | 20 ml |
| Organic acids stock | 10 ml |
| Casein Hydro lysate | 500 mg |
| Glucose | 33.7 g (0.17 M) |
| Mannitol | 30.92 g (0.17 M) |
| BSA | 1 g |
| NAA | 1 mg |
| BAP | 0.4 mg |
| pH | 5.6 (KOH) |
| *filter sterilize |  |

| **Alginate solution*** | **For 500 ml** |
| --- | --- |
| Alginic acid-Na salt | 14 g (2.8% (w/v)) |
| Sorbitol | 36.44 g (0.4 M ) |
| * autoclave and store at 4°C | |

| **Setting agar*** | **For 1 L** |  |
| --- | --- | --- |
| Sorbitol | 72.88 g (0.4 M) |  |
| CaCl_2_ · 2H_2_O | 7.351 g (50 mM) |  |
| Phyto agar | 8 g |  |
| * filter sterilize in ½ of the final volume, then add to autoclaved agar in the other half | | |

| **Floating solution*** | **For 1 L** |
| --- | --- |
| Sorbitol | 72.88 g (0.4 M) |
| CaCl_2_ · 2H_2_O | 7.351 g (50 mM) |
| * filter sterilize | |

| **Medium F*** | **For 1 L** |
| --- | --- |
| MS modif. No. 4 (Duchefa) | 2.70 g |
| NH_4_Cl | 107 mg |
| Vit. NN stock | 1 ml |
| Adenine sulphate | 40 mg |
| Casein hydrolysate | 100 mg |
| Sucrose | 2.5 g |
| Mannitol | 54.7 g |
| NAA | 0.1 mg |
| BAP | 0.5 mg |
| pH | 5.8 (KOH) |
| *filter sterilize | |

| **Releasing solution*** | **For 100 ml** |
| --- | --- |
| Na-Citrate | 588.2 mg (20 mM) |
| Sorbitol | 9.11 g (0.5 M) |
| * filter sterilize |  |

| **Medium G*** | **For 1 L** |
| --- | --- |
| MS modif. No. 4 (Duchefa) | 2.70 g |
| NH_4_Cl | 267.5 mg |
| Vit. NN stock | 1 ml |
| Adenine sulphate | 80 mg |
| Casein hydrolysate | 100 mg |
| Sucrose | 2.5 g |
| Mannitol | 36.4 g |
| IAA | 0.1 mg |
| Zeatine | 2.5 mg |
| pH | 5.8 (KOH) |
| * filter sterilize | |

| **Medium H *** | **For 1 L** |
| --- | --- |
| MS salts and organics (Duchefa) | 4.4 g |
| Sucrose | 10 g |
| Zeatin | 2 mg |
| NAA | 0.01 mg |
| GA_3_ | 0.1 mg |
| Gelrite (Duchefa) | 2.5 g |
| pH | 5.8 (KOH) |
| * filter sterilize in ½ of the final volume, then add to autoclaved gelrite in the other half | |

| **Medium I*** | **For 1 L** |
| --- | --- |
| MS salts and organics (Duchefa) | 4.4 g |
| Sucrose | 20 g |
| GA3 | 0.1 mg |
| Gelrite (Duchefa) | 2.5 g |
| pH | 5.8 (KOH) |
| * filter sterilize in ½ of the final volume, then add to autoclaved gelrite in the other half | |

| **Vitamins MS stock*** | **For 50 ml** |
| --- | --- |
| Thiamine-HCl | 5 mg |
| Pyridoxine-HCl | 25 mg |
| Nicotinic acid | 25 mg |
| Glycine | 100 mg |
| Myo-Inositol | 5000 mg |
| * filter sterilize. Store aliquots at -20°C | |

| **Macro stock*** | **For 1 l** |
| --- | --- |
| KNO_3_ | 74 g |
| MgSO_4_ · 7H_2_O | 49.2 g |
| KH_2_PO_4_ | 3.4 g |
| * filter sterilize. Store at 4°C | |

| **CaCl_2_ stock*** | **For 100 ml** |
| --- | --- |
| CaCl_2_ · 2H_2_O | 29.4 g (2M) |
| * filter sterilized. Store at 4°C | |

| **Iron stock*** | **For 100 ml** |
| --- | --- |
| Na_2_EDTA | 140 mg |
| FeSO_4_ · 7H_2_O | 190 mg |
| * filter sterilized. Store at 4°C | |

| **Micro stock*** | **For 100 ml** |
| --- | --- |
| H_3_BO_3_ | 150 mg |
| MnSO_4_ · H_2_O | 500 mg |
| ZnSO_4_ · 7H_2_O | 100 mg |
| Na_2_MoO_4_ · 2H_2_O | 12 mg |
| CuSO4 · 5H_2_O | 1,2 mg |
| CoCl_2_ · 6H_2_O | 1,2 mg |
| KI | 38 mg |
| * filter sterilized. Store at 4°C | |

| **Vit. mix 1 stock*** | **For 100 ml** |
| --- | --- |
| Pantothenoic acid | 50 mg |
| Choline Chloride | 50 mg |
| Ascorbic acid | 100 mg |
| p-Aminobenzoic acid | 1 mg |
| Nicotinic acid | 50 mg |
| Pyridoxine-HCl | 50 mg |
| Thiamine-HCl | 500 mg |
| * filter sterilize. Store aliquots at -20°C | |

| **Vit. mix 2 stock*** | **For 100 ml** |
| --- | --- |
| Folic acid | 20 mg |
| Biotin | 0,5 mg |
| Cyanocobalamin (Vit. B12) | 1 mg |
| * filter sterilize. Store aliquots at -20°C | |

| **Vit. mix 3 stock*** | **For 100 ml** |
| --- | --- |
| Cholecalciferol (Vit. D) | 0.5 mg |
| * filter sterilize. Store aliquots at -20°C | |

| **Sugars stock*** | **For 100 ml** |
| --- | --- |
| Sorbitol | 625 mg |
| Sucrose | 625 mg |
| D(-)Fructose | 625 mg |
| D(-)Ribose | 625 mg |
| D(+)Xylose | 625 mg |
| D(+)Mannose | 625 mg |
| L(+)Rhamnose monohydrate | 625 mg |
| D(+)Cellobiose | 625 mg |
| Myo-Inositol | 250 mg |
| * filter sterilized. Store at 4°C | |

| **Organic acids stock*** | **For 100 ml** |
| --- | --- |
| Pyruvic acid | 100 mg |
| Fumaric acid | 200 mg |
| Citric acid monohydrate | 200 mg |
| DL-Malic acid | 200 mg |
| * filter sterilized. Store at 4°C | |

| **Vitamins NN stock*** | **For 50 ml** |
| --- | --- |
| Glycine | 100 mg |
| Myo-Inositol | 5000 mg |
| Thiamine-HCl | 25 mg |
| Pyridoxine-HCl | 25 mg |
| Nicotinic acid | 250 mg |
| Folic acid | 25 mg |
| Biotin | 2.5 mg |
| * filter sterilize. Store aliquots at -20°C | |

Y. P. Wang

2

, K. Sonntag

1,4

, E. Rudloff

1

and J. Han

3

Y. P. Wang

2

, K. Sonntag

1,4

, E. Rudloff

1

and J. Han

3
